# Work-related challenges and their associated coping mechanisms among female head porters (Kayayei) in Ghana

**DOI:** 10.3389/fpubh.2024.1383879

**Published:** 2024-07-17

**Authors:** Joyce Komesuor, Emmanuel Manu, Anna Meyer-Weitz

**Affiliations:** ^1^Department of Population and Behavioral Sciences, University of Health and Allied Sciences, Ho, Ghana; ^2^School of Applied Human Sciences, University of KwaZulu-Natal, Durban, South Africa

**Keywords:** migrant workers, job demands and resources, informal workers, Kayayei, physical health and well-being, resilience, mental health

## Abstract

**Background:**

While internal migrants (Kayayei) in Ghana have been perceived as a vulnerable group facing various health-related challenges, there has not been enough research on the impact of their work on their health and well-being. This study investigated the lived experiences of the Kayayei to identify the health-related challenges associated with their work and the coping mechanisms they adopt in dealing with these challenges.

**Methods:**

We interviewed 21 participants purposely selected and conducted two focus group discussions (FGD) of five participants each at the Agbogbloshie market. Interpretive Phenomenology Analysis Approach was used to identify themes and sub-themes. Statements from participants were presented as quotes to corroborate their views.

**Results:**

The work-related challenges identified in the study were physical health, mental health, accommodation, and social challenges. Religion, recreation, social support, hope, resilience, and self-medication were the coping strategies adopted by the study participants.

**Conclusion:**

The government of Ghana should be encouraged to work with stakeholders like social welfare to raise awareness about women’s rights, build their skills to increase their employment opportunities, enhance their safety, health, and overall well-being. It is also important to ensure the networking of relevant stakeholders to work with women in the informal sector to foster agency and provide support when needed.

## Background

Internal migration is a global phenomenon due to its importance in the socio-economic development of individuals and nations (1). Hence, when internal migration is properly managed, it is a strong force for prosperity and progress (2). For instance, in China, the tremendous number of rural workers who moved to cities due to huge income disparities between the rural and urban communities significantly contributed to China’s recent economic development (3). Furthermore, studies have shown that countries that attracted internal migrants more than a century ago are much richer today, emphasizing the long-term economic impact of internal migration (4).

In Sub-Saharan Africa, migrant workers often move from rural communities where they usually engage in subsistence agriculture or other menial jobs in cities in search of greener pastures (5). These movements often lack proper coordination and planning, leaving the migrants frustrated and vulnerable upon arrival in their host cities (6). Thus, they are often faced with increased health hazards due to institutional barriers to health such as social exclusion, discrimination, and unfavorable working and living conditions (7). The situation is often dire for female migrants who become vulnerable to various forms of exploitation including sexual harassment and trafficking as they learn to navigate their new environments (8).

Ghana has witnessed rapid urbanization in the last few decades due to the movement of people from rural to urban centers in search of better standards of living (9). According to the Ghana Statistical Service (GSS) (10), the urban population of Ghana has increased from 50.9 percent in 2010 to 56.7 percent in 2021 mainly owing to internal migration. A key category of people who are an integral part of internal migration in Ghana is the “Kayayei.” Kayayei is a Ghanaian term for female head porters who help shoppers carry goods to their destinations, mostly in the cities (11). These Kayayei are mostly unskilled and in informal and unprotected work, they often engage in hazardous jobs that severely affect their physical and mental well-being (12). They face challenges such as access to food, shelter, lodging, safety and security (13–15).

Although Ghana continued to experience steady marginal economic growth, inequality has widened, and poverty remains prevalent, especially in the northern regions (9). There are higher levels of poverty, unemployment and infrastructural deficit in the north as opposed to the south, as a result of neglect by successive governments (16). This influence a number of the Kayayei to migrate from the north to the south in pursuit of better economic opportunities (17). Some also migrate due to cultural and religious oppression such as forced or child marriages (18). However, in many instances, the hope for better economic opportunities is not realized for the Kayayei and they are therefore compelled to resort to precarious informal work arrangements. Informal and unregulated work arrangements are known to have little or no protection implying that the demands are likely to exceed the available resources (19).

As posited by Bakker and Demerouti (19), every occupation has its demands that may constitute stressors that affect the well-being of workers but at the same time, have resources that are leveraged to moderate these stressors. For instance, high work pressure, an unfavorable physical environment and emotionally demanding interactions with clients or colleagues, all constitute job demands. The various organizational support and personal resources, e.g., self-regulation and other coping strategies often employed by workers to address job demands are viewed as job resources (19, 20). While job hindrances and challenges may differ from one individual to another, even in the same working environment, they often affect the health and well-being of all workers (19, 20). For the informal and vulnerable worker, job resources would seem to be the primary responsibility of the individual in the form of psychological and social support resources. For instance, a study in India by Ray (21) among even well-educated GiG workers, social networking and support was found necessary to mitigate job demands. Further, a recent report from the International Labor Organization (ILO) on informal work arrangements among women argues for collective agency in managing job demands and seeking support (22). It is however important to identify and report the various job challenges and hindrances that may affect the health and well-being of the Kayayei and the job-related resources they leverage to mitigate these demands and stressors. Hence, the provision of an enabling environment and conditions to mitigate these challenges and hindrances is paramount in ensuring the physical, mental and emotional health and well-being of workers (23).

Although the plight of the Kayayei has been reported in the literature, they remain a vulnerable and underserved population in terms of scholarly reporting. For instance, studies on internal migration have mostly concentrated on its economic impact on the lives of migrants and their families back home (24–27). Some studies have identified priority areas for migrants for health promotion action (28, 29) and imparting internal migration experiences, health, and well-being issues among Kayayei in Ghana (30, 31). However, these studies have not delved into the self-reported coping strategies of the Kayayei regarding the challenges they face. Thus, the narrative is incomplete if only Kayayei’s experiences and challenges are reported, but no understanding of how they cope amidst the challenges the face. Moreover, the plight of the Kayayei needs to be holistically understood to develop interventions and policies that could improve their general health outcomes as per the mandate of the Sustainable Development Goals (SDG) which advocates for better health for all and also urges governments to facilitate orderly, safe, regular, and responsible migration and mobility of people by 2030 (32).

Against this background, we explored the lived experiences of the Kayayei to identify the health-related challenges associated with their work as well as the coping mechanisms they adopt in dealing with these challenges. This study further endeavored to provide the necessary information to support informed decision-making to improve the health and well-being of the Kayayei.

## Materials and methods

### Study design

We used an Interpretative Phenomenological Analysis (IPA) design for the qualitative in-depth interviews and focus group discussions. This approach enables the description of participants’ detailed lived experiences and the identification of common themes among them (33, 34). IPA does not generate accounts based on pre-existing theoretical preconceptions that have already been prescribed, but rather the accounts are generated based on the lived experiences on their terms. According to Neuman, this approach allows individuals to create meanings in their worlds (35). These meanings are created through interaction with other people within their environment. Although the IPA focuses on how individuals make meanings of their words, the research is dynamic, with the researcher taking an active role in the process by getting close to the participants’ world (36). The phenomenon being examined in this study is how the lived experiences of Kayayei impact their mental and physical health. Thus, using an IPA enabled us to capture descriptions and meanings that a group of Kayayei had about their experiences of being Kayayei. To meet the requirement for IPA, we conducted interviews and focus group discussions among the Kayayei and analysed data based on the ‘what’ and ‘how’ of participants’ experiences (34, 37).

### Participants and sampling

Participants for the study consisted of 21 Kayayei for in-depth interviews and another 10 Kayayei for focus group discussions recruited from the Agbogbloshie Market in Greater Accra, the Capital of Ghana. We used purposive sampling to select participants for the study. Purposive sampling is often used to select participants based on preselected criteria relevant to a particular subject matter being studied which will provide important information on the phenomenon being investigated (38, 39). For inclusion in the study, the participant had to have been a Kayayei, be operating at the Agbogbloshie market, be in Accra for more than 6 months, and be 18 years and older. We did not predetermine the sample size for the study, we however reached saturation by the 21st participant for the in-depth interviews. In qualitative research, saturation is reached when no new information is added to those that previous participants have already provided as outlined by Braun and Clarke (40). Two groups of five new participants were selected for focus group discussions (FGD). FGDs allow for the development of knowledge and co-constructed meanings at the group level (41, 42). FGD also helps to ascertain whether participants behave differently when they are in a group setting as compared to the one-on-one interview with the researcher. The study selected Kayayei from different age groups and experiences to share their lived experiences on the Kayayei business to generate broad overviews of the phenomenon. The combination of FGDs and individual interviews gave clearer insight into the lived experiences of the Kayayei (43–46).

### Data collection procedure

The Kayayei were approached through the leadership of the Kayayei Association to participate in the study. The president of the Kayayei Association called them for a meeting and allowed the researchers to explain the purpose of the meeting to them. After the detailed explanation about the aim of the study, a few declined to take part but most of them agreed to take part on a convenient day. Phone numbers of those who were not available were collected and were subsequently called and those who were reached through the phone were briefed on the purpose of the study. After participants agreed to take part in the study, the date, time, and locations that were most convenient to them were decided on for the interviews.

### In-depth interviews

A semi-structured in-depth interview guide was used to guide the interviews aligned to the objectives of the study. Semi-structured interviews offer a framework while giving the interviewer flexibility to elicit more information or seek clarification using follow-up questions or probes (47). While some key questions appeared in the interview guide, it was flexible enough to allow the researchers to probe with follow-up questions. The interview guide had three sections. Each section has major questions with several probes to obtain further clarification and follow-up. Interviews were conducted predominantly in English since 71.4% of participants had high school education, the rest were conducted in Twi and Sisala. The interviews were conducted by the first author and two research assistants with support from interpreters. Data was collected between July and August 2018. With permission from participants, the interviews were audio recorded. The interviews lasted between 1 h-to-1 h 20 min.

### Focus group discussions

FGDs are discussions that are organized to explore a specific set of related issues and experiences (48). The collective activity of the group provides added dimensions of the interaction among members (48, 49) In this study, the FGD was facilitated by the first author and one research assistant using an FGD guide. The FGD guide consists of three sections including background characteristics, work-related challenges, and coping strategies. The purpose of the FDG is to ascertain whether participants behave differently when they are in a group setting as compared to the one-on-one interview with the researcher. The FGD discussions lasted between 1 h 30 min to 2 h.

### Data analysis

The audiotapes were transcribed verbatim by two research assistants with the exact details about voice intonation and meaning. Steps were taken to minimize bias by using multiple people in the coding process, checking for alternative explanations, and reviewing the findings with peers as suggested by Giorgi (47). The interviews conducted in English were transcribed by playing the audio recordings over and over to make sure nothing was missed. Interviews conducted in the other languages were transcribed into English. The analysis followed the guidelines stipulated in IPA as outlined by Storey (48) in three steps. Step one entailed initial reading and re-reading of transcripts to identify initial codes which were combined to form sub-themes within the data. In step two, the individual themes were linked to create thematic clusters. Step three involved the creation of a thematic table that had various themes.

The first Author and the two research assistants reviewed the transcripts several times and identified emerging themes titles and transforming them into phrases that captured themes found in the transcripts (35). Emerging themes were listed using the questions in the interview guide, while connections between them were identified and patterns of meaning across the datasets were made to provide in-depth understanding. Patterns were identified through a thorough process of data familiarization, coding, and theme development and revision. The authors then identified thematic clusters and subordinate concepts. In the third stage, a summary table was created to illustrate the emerging main themes and sub-themes identified with quotes to substantiate the issues as suggested by Clarke and Braun (49). The reliability of qualitative data is determined by the extent to which data is the correct representation of the variables measured (50). For the data to be reliable, the lead author and one coder independently assessed the reliability of the coding process by applying the themes and sub-themes to each participant’s transcript. The coders indicated (0) when a particular theme is absent and (1) when the theme is present. We calculated the agreement between the coders by the number of times they agreed on each sub-theme. The overall interrater agreements ranged between 85 to 100%. We, therefore, determined that our reported data is the actual representation of the variables measured.

## Results

### Background characteristics of participants

Table 1 presents the socio-demographic characteristics of participants. The findings show that the oldest participant was 40 years old while the youngest was 18 years. The findings further indicated that 38.1 percent (n = 8) of the participants had completed Senior High School (SHS). Most of the participants 19 (90.5%) were Muslims, while the majority 8 (38.1%) were Mamprusi.

**Table 1:** Background characteristics of the participants.

Characteristics	In-depth interviews with Kayayei		Focus group discussions with Kayayei	
	Frequency [*N* = 21]	Percentage [%]	Frequency [*N* = 10]	Percentage [%]
Age				
18–19	5	23.7	3	30
20–29	14	66.7	4	40
30–39	1	4.8	2	20
40+	1	4.8	1	10
Mean age				
Education				
No formal education	3	14.3	2	20
Primary	3	14.3	2	20
JHS*	7	33.3	3	30
SHS*	8	38.1	3	30
Marital status				
Single	11	52.4	6	60
Married	10	47.6	4	40
Ethnicity				
Sisala	6	28.6	3	30
Dagomba	7	33.3	2	20
Mamprusi	8	38.1	4	40
Religion				
Muslim	19	90.5	9	90
Christian	2	9.5	1	10

*SHS, Senior High School; JHS, Junior High School.

#### Findings

We triangulated the findings of the interviews and focus group discussions, and the outcome of the results converged thereby integrating the findings, as depicted in table 2. The triangulation enabled us to develop a comprehensive understanding of the phenomenon under study. Two major themes were realized. These were work-related challenges and coping strategies and resources for everyday life. Sub-themes were further identified within themes, the primary work-related challenges, i.e., job demands were; physical health, mental health, accommodation, and social challenges. Sub-themes for coping strategies, i.e., resources, were religion, recreation, social support, self-medication, and resilience.

**Table 2:** Themes and sub-themes.

Theme	Sub-Theme	Code (frequency)
Work-related challenges	Physical health challenges	Body pains and fatigue (15) Falls leading to injury (3) Motorcycle and vehicular accidents leading to death or injury (4) Falling due to weight of load and fatigue (5) Effect of work on overall physical health (11)
	Mental health challenges	Stress [difficulty to relax, agitation] (4) Anxiety [frequent trembling in hands, loud heartbeats, fear without reason] (8) Depression [not feeling content, suicidal thoughts, frustration] (5)
	Accommodation challenges	Overcrowding (10) Bed bugs (7) Insecurity (12) No accommodation (use storefronts with mosquito net) (3)
	Social challenges	Sexual and physical harassment (8) Sexual assault (3) Negative attitudes of clients (9)
Coping mechanisms	Religion	Hope in God (8) Prayer (5) Refuge in moral values of religion (4)
	Recreation	Listen to music or watch movies (3)
	Social support	Support from friends (5)
	Resilience	Patience and self-control (4) Self-care
	Self-medication	Frequent intake of pain medication (5)

### Work-related challenges faced by the Kayayei

The participants in the study shared their typical working day challenges and demands that posed stressors to their health and well-being. In this regard, four sub-themes emerged, i.e., physical health, mental health, accommodation, and social challenges.

#### Physical health challenges

With regards to the physical nature of the Kayayei work, they mentioned that their work exposes them to all kinds of injuries through motorcycle and vehicle accidents as well as falling due to the weight of the load while walking long distances resulting in fatigue. Others also mentioned regular body pains and frequent headaches.

##### Motorcycle and vehicular accidents

Four participants explained that carrying heavy loads along or across the busy roads of the city puts them at risk of being knocked over by speeding vehicles and motorcycles. Some of their narratives are captured in this quote:

*We face dangers at work. At the roadside, a motorbike or car can hit you. Someone can get hit and she will get injured or she will die. The riders do not stop when they hit you. If you are lucky and nothing happens to you then that’s good*. (Single, 22 years old)

##### Falling due to weight of load and fatigue

With regards to falling due to the weight of the load and fatigue being a danger associated with the work of Kayayei, five participants explained that sometimes, due to the weight of the load, distance covered with the type of load and efforts to avoid getting knocked down, they lose their balance and fall. These falls sometimes lead to muscle strains that result in pain and wounds which impact negatively their ability to work. The direct quotation is as follows:

*There are times when the load gets so heavy that one even falls with the load. If something bad happens to the load in the process, the owner asks us to pay for the damages*. (Married, 25 years old)

##### Body pains and fatigue

Regarding body pains, it was shown that 15 participants experienced pains in the neck, backache, legs, and joints as a result of the strenuous work of carrying heavy loads, climbing footbridges, and walking long distances with such loads. A 22-year-old single Kayayei stated, “*Sometimes the load can make one fall and break one’s leg. Sometimes, the fall also causes pain in my chest.”* Furthermore, another 20-year-old single Kayayei narrated “*I get very weak as a result of working. I feel pains in my legs, backbone, thighs, and arms*”.

##### Falling leading to injury

Concerning falls leading to injury, four Kayayei narrated that they had experienced a fall and injured themselves due to the heavy load they carried, or they had at times been pushed by motorcycles or vehicles causing them injuries. Some of their views are captured in the following quote:

*The work is very risky. Sometimes you could carry something and fall. Last week, for instance, I fell into the gutter and injured myself*. (Single, 22 years old)

##### Effect of work on overall physical health

Eleven participants also expressed frustration at the effect of the work on their overall physical health. Some of them stated that the difficult nature of the work can make them so sick to the extent that they will be absent from work for months or their strength will be reduced making it difficult to work. They consequently attribute this to negative physical health outcomes such as chest pains and difficulty sleeping. The following quote summarizes their views:

*For me, it’s my chest that’s paining me. For more than three (3) weeks in the last month I have not come to work because of my chest pain. It makes it very difficult for me to breathe. It’s like something is pressed on my heart when I try to breathe* (Single, 22 years old)

#### Mental health challenges

It seems that the participants have experienced stress, anxiety, and depression. The Kayayei explained that the nature of their work and the treatment by some of their patrons resulted in them experiencing mental distress. The study participants talked about their experiences of mental health distress.

##### Depression

The Kayayei spoke about the daily fluctuations of their moods from happiness to despondency and depression. While some of the Kayayei said they were always happy, five of them stated that they were unhappy, and some were not feeling content with their lives. Those who were not always happy explained that their happiness is affected by financial issues of the day, i.e., whether they have made enough money or not. For instance, a forty-year-old married Kayayei stated “*my happiness is mixed. For instance, I can be happy today but not tomorrow when I do not get anything out of my work*”.

Those who were not content with their lives said they were not happy with the life they led and particularly because of the conditions under which they lived and the situation they found themselves in. It seems that not being in a position to make some money to make the daily sacrifices they endure worthwhile and being unable to return home is at the core of their despondency. Below are a quotes that reflects their emotions:

*If I say I am happy, I would be lying. It’s money that we are working to get and once we get the money, we will be happy in our lives. If we were living in our hometowns, we would not be going through some of the things we are going through here* (Married 25 years old)

*I come to market to work to make money, and after going around the whole day and coming home with virtually nothing, I lie down and cannot sleep I think about this life I am leading now. How do I feed my children? (Married, 40 years old)*

Some participants experienced suicidal thoughts and explained that this is often triggered by daily events. It is then that they sometimes wish that they had never been born and feel like ending their lives. This was particularly noted among the younger women. They have however shared that they have never attempted suicide before. A 22-year-old single participant stated “*Sometimes when something bad happens to me, I ask “God what wrong have I done to deserve this?” I feel like if death was being sold, I would have gone to buy it and die.*

##### Anxiety

It seems that eight participants do experience symptoms that could be related to anxiety**. S**ome participants experienced trembling hands, rapid heartbeats and felt fearful without obvious reasons. The following quotes relate to their experiences:

*I will be going about my normal day-to-day activities and suddenly, my heart will start pounding like ‘fufu’.* (Married, 40 years old)

*I will just sit and feel fear although I know no one is coming to do anything to me.* (Single, 22 years old)

*For me, I experience trembling in my hands like twice a week and I do not know why it happens to me*. (Single, 22 years old)

##### Stress

Four participants stated that due to the nature of the Kayayei business, they are always stressed because of the abuse by some customers and at times not getting enough money for the day to even buy food to eat. The struggle for daily livelihood brings great uncertainty and is thus very stressful.

*I come to market to work to make money, and after going around the whole day and coming home with virtually nothing, I lie down and cannot sleep I think about this life I am leading now. How do I feed my children?* (Married, 40 years old)

#### Accommodation challenges

Challenges around accommodation contributed to daily struggles for the Kayayei. The quotes derived from the issues of accommodation include overcrowding, bed bug infestation, insecurity, and no accommodation.

##### Overcrowding

Ten participants explained that the accommodations were predominantly wooden stores and are mostly overcrowded with as many as 8–13 Kayayei having to share a space/room. Most notable is the fact that the accommodation does not come with toilet facilities. Below is a quote from a participant,

*We are 10 in a room and each person pays 5 cedis, about ($1) each week. The roof of where we sleep leaks, and the ground gets wet when it rains. We pay separately to access the toilet and bath*. (FDG 1, single, 18 years old)

##### Bed bug infestation

Seven participants added that their accommodation is infested with bed bugs, forcing them to sleep outside most of the time. An 18-year-old single Kayayei stated, “*There are bed bugs and rats there. So, we mostly sleep outside.”* Below is a response from another participant.

*Accommodation is not good at all. About eight (8) to ten (10) people sleep in one room. Rain and mosquitoes disturb us so much. There are rats and cockroaches in the room, but we do not have any other choice but to stay there*. (Married, 33 years old)

##### Insecurity

There is little security in these places, seven participants indicated that they are sometimes attacked by armed robbers or have their savings stolen by thieves or even fellow Kayayei. Below is a quotation to sum up their views.

*Since there are many in the rooms we stay, there are theft issues and no one owns up when a person complains about a missing item. Now we save our money on MTN so the stealing has come down a little*. (Single, 22 Years Old)

##### Lack of accommodation

For participants without accommodation, three participants explained that they sleep in front of shops at night when they are closed. In some cases, they had to pay tokens to the shop owners for sleeping in front of their shops after opening hours or sweep the front of the shops early in the morning before opening hours as a way of payment.

*I sleep here (points to a shop) where the yellow carpet is. In the night we fix the mosquito net and use a large black rubber to cover the whole place, light some mosquito coils and we sleep inside. Every week, we pay 5 cedis ($1) each. When we wash our clothing and put them on the drying line to dry, they get stolen. Other belongings such as soaps, sanitary pads, money and many other things also get stolen, sometimes by our colleagues*. (Single, 20 years old)

#### Social challenges

The Kayayei are vulnerable to sexual abuse, physical assault, and verbal abuse. They are at risk of being sexually exploited just to make ends meet.

##### Sexual and physical harassment

There are issues of sexual and physical harassment by male clients. Eight participants mentioned that they had experienced sexual harassment by male clients

*Men like touching the Kayayei. Sometimes, you carry load to the station and the ‘loading boys’ touch you and tell you how nice you are. Anytime someone tries that, I warn the person and leave because I don’t know them*. (Single, 22 years old)

##### Sexual assault

Due to their vulnerability, these Kayayei as well as their female children become victims of sexual assaults by predators in society. Three participants mentioned that they have either been sexually assaulted or seen someone who has experienced sexual assault.

*As we sleep outside, we are prone to rape. Recently, a child was raped. The rapists have some chemical that they spray into the air that causes us to sleep very deeply and they rape some people even before other people wake up*. (Married, 33 years old)

##### Negative attitudes of clients

Nine participants in this study further expressed their frustration at the verbal abuses they endure at the hands of their patrons and the public at large. They explained that due to their vulnerability in society, they are sometimes subjected to verbal and physical assault by clients for the smallest misunderstanding or when breaking goods because of a fall. The following are some of the expressions by the Kayayei:

*Sometimes after offering your services to someone, the person can verbally assault you, and if you reply, the person can beat you up because there is no one to report to*. (Single, 22 years old)

### Coping strategies

The Kayayei use various coping strategies such as taking refuge in religious teachings, engaging in recreational activities, and seeking social support from family and friends as well as supporting each other as job resources at their disposal to mitigate job demands. Some participants mentioned using multiple strategies to cope with the problems associated with their work as head porters.

#### Religion

From the findings, it is clear that religion is the primary source of coping with daily living and finding solace and hope. Their resilience is also evident from the positive emotions and hope they experience despite acknowledging their daily challenges. Religion also seems to be an important coping strategy as many participants explained that they put all their hope and trust in God in their prayers, which helps them cope with challenges related to their work. Eight participants indicated that their hope in God for a better future sustains them in dealing with the challenges of life. A 40-year-old married Kayayei expressed her opinion in the following quote “*I take my support from God. We do not really have a support group.”* Below are some of the quotes from the participants:

*Whenever I don’t get any money after a hard day’s work, I just go home and look up to God for help. All I do is just pray with the hope that things will change*. (Married, 22 years old)

Prayer also seems to play a significant role in how the Kayayei cope with their daily lived experiences. Five participants mentioned that they always pray when things become difficult instead of using maladaptive coping strategies such as alcohol and other drug use. Below is a quotation that sums up their narratives

*Being a Christian has been beneficial to me because I don’t have to indulge in alcoholism to forget my problems. If things get difficult, I pray to God to help me against sickness and excess anger*. (Married, 22 years old)

### Recreation

Recreational activities are used as coping mechanisms, they participate actively used recreational activities to reduce specific stressors related to their activities. Three participants said they engage their friends in conversations and listen to music or watch movies to cope with challenges associated with their work. For instance, a 19-year-old Kayayei stated, “*I join people who are watching movies and laughing.* Some of their narratives are captured in the following quotes:

*if things happen to me, I always go and sit by someone who is playing music or with my sister and friends and these take my thoughts and worries away from the problem*. (Single, 18 years old)

### Social support

This study also found that the presence of social support helped the Kayayei to cope with their situation even though only a few of them stated that they had no social support.

Five participants revealed that friends play an important role in helping Kayayei manage their difficulties through words of encouragement. For instance, a single 18-year-old participant put it this way, *“I have a friend so anytime I am in difficulty, I call her”*. A 26-year-old married participant put it this way, *“I stay among friends, and when we converse, I get back happy*.

### Resilience

In narrating their daily struggles and coping strategies, it was evident that the Kayayei demonstrated some level of resilience which enabled them to persevere in the difficult situations they find themselves in.

Participants demonstrated resilience through patience and self-control. Four participants shared their positive emotions, optimism and hope for each day:

*I know that even though the work I am doing is very difficult, I know my life will change if there is a long life, so I take it just like that*. (Single, 22 years old)

It seems that a few of the participants use self-regulating practices for self-care as a way to cope by deliberately involving themselves in activities that enhance their emotional, mental, and physical health, thus to destress. One participant shared her self-care strategy:

*At times my health will not allow me to work, or a client will make me angry, I just sleep and do not go to work that day, and when my friends ask for the reason, I tell them I came to look for money and not money looking for me, so I will take things easy*. (Married, 29 years old)

### Self-medication

Five participants noted that due to the physical nature of the Kayayei business and the frequent physical pain they experience, they often resort to self-medication to cope with the pain.

Instead of going to the health facilities for proper diagnoses, most of the participants rely on painkillers to relieve them of the constant pain they experience. Below is a quote that summarizes their narratives:

*I get tired from work to the extent that I sometimes cannot wake up from bed. The only thing that can enable me to work the following day is the use of drugs. The work sometimes makes me very dizzy*. (FDG 1, Married, 20 years old)

*When the pain becomes unbearable, I contact this guy who sells a mixture, people say it contains ‘wee’ (Marijuana), but I do not know what is inside, I only take it to relieve the pain I am feeling*. (Single, 18 years old)

## Discussion

This study explored the lived experiences, health-related challenges and the associated coping mechanism of internal migrants in Ghana. Participants narrated their lived experiences and the mitigating coping strategies they adopted.

### Daily work-related challenges

Individuals have different reasons for migrating to the city areas with the most common reason being the quest to earn an income to sustain their own and families’ livelihoods (51–53). This is no different for the Kayayei, however, female migrants might have additional stressors in their quest to earn a sustainable income (54, 55). The work-related challenges viewed as job demands, in the absence of adequate resources, pose major stressors to the Kayayei. These seem to impact their physical health and well-being, as suggested by the JD-R model (19, 20).

The study found that the physical nature of the work exposes the Kayayei to various physical health challenges, e.g., injuries through motorcycles and vehicle accidents, buckling under the weight of the load and fatigue due to walking long distances. These result in backache, pains in the neck, chest, legs and joints due to the strenuous work of carrying heavy loads over long distances, similar to findings reported in previous studies (11, 56, 57). The alleviation of these physical symptoms seems to diminish some of the daily earnings of the Kayayei as they spend their money on buying medication to mitigate the pain and enable them to manage the job demands of the next day. The seemingly high prevalence of self-medication or even other illegal drug abuse among the Kayayei is of concern, especially as they appear unable to access regular medical care from qualified health professionals. Aspects such as time constraints and affordability of basic health care are common problems of informal workers as indicated by Sánchez (58). This study’s findings are corroborated by a previous study of Yeboah (59), where the poor working conditions, coupled with little rest and not earning enough income, put the Kayayei at risk of various physical health conditions. Furthermore, the incidence of abuse, exploitation, and working under unsafe working conditions have been found to negatively impact the physical health of informal workers in Ghana (13, 31).

According to the World Health Organization (WHO) (60), mental health is an essential part of an individual’s ability to lead a fulfilling life, i.e., the ability to form and maintain relationships, study, work, and make other important decisions in life. Factors that impact mental well-being thus impede the optimal functioning of not only the individual but also have negative outcomes for the family and the society at large (60). The mental health challenges reported by the Kayayei is suggestive of depression, anxiety, and stress. Other studies have also reported mental health challenges among migrant workers, e.g., Li and colleagues (61) reported higher levels of depression among migrants than non-migrants in Shanghai. Similarly, mental health challenges among migrants were also reported by Yang et al. (62) and Komesuor and Meyer-Weitz (13).

It has been argued that the migration process predisposes migrants to mental health distress in various ways. The change in socio-cultural settings (63–65) often lead to a loss of cultural identity and difficulty adapting to the new social norms as well as navigating the local environment (51, 66). The disruptions of the normal social norms and networks may have a profound effect on the mental health of the Kayayei as observed by Kirmayer et al. (67). However, it is difficult to imagine and measure the costs of loss in friendships, family, and social networks, and the difficulties in establishing new relationships in places where the societal norms are very different (51, 68). The extent of the mental health challenges among the Kayayei could also be due to discrimination and perceived social inequity they witness in their host cities, primarily as a result of being women (68–70). The lack of national policies regulating and protecting the rights of informal workers, particularly female workers, and other self-employed individuals in Ghana (71), is an issue that is of critical importance to be addressed within Ghana and elsewhere.

Migration may therefore result in isolation, a lack of money and other socio-economic challenges including a lack of accommodation in the new host community (7). The lack of accommodation in general and particularly decent accommodation, was highlighted by the Kayayei in this study as a source of distress and anxiety. Previous studies have also reported that the Kayayei find it difficult to obtain affordable accommodation upon their arrival in their host cities (11, 72, 73). This reality forced many participants to find shelter in front of Kiosks with little or no security nor safety as they reported being attacked by armed robbers, sexual violence including abduction by predators.

The Kayayei in this study reported experiencing sexual and physical harassment, sexual assaults, as well as discrimination and negative attitudes of clients. This confirms previous findings of sexual violence against female migrant workers (74, 75). Maltreatment and discrimination against migrant workers have also been reported in various other studies (76, 77). It should also be noted that migrant women who engage in informal work might be more vulnerable to abuse and maltreatment because they often hold jobs for which there is little protection under social legislation (78, 79). It seems that in most countries, women do not have the same rights and opportunities for employment as men do, while they are often also expected to take responsibility for the survival of the whole family by searching for sources of income, no matter the circumstances (80, 81). While all these challenges and experiences may explain Kayayei’s mental distress, they also self-regulate to de-stress and demonstrate resilience by remaining committed to finding ways to cope in their quest for a livelihood.

### Coping strategies

The Kayayei adopted multiple coping strategies, which served as important personal resources to mitigate the job demands they faced and enhance their resilience to continue working. Bakker and De Vries (23) integrated perspectives of adaptive self-regulation in the theory underlying the JD-S in combination with personal resources as critical in buffering against job strains and thus burnout. The study findings show how the Kayayei employ various strategies to cope with the adversity they experience in daily work. These include reliance on their religion that gives them hope for a better life, engaging in self-regulation strategies such as recreational activities and self-care practices; self-medication to continue work despite the pain; seeking and giving social support to their fellow Kayayei and demonstrating resilience in managing their daily lived experiences while remaining committed to their goals of earning an income.

The Kayayei self-regulate their work strain through self-care by engaging in recreational activities such as listening to music or watching movies. The study by Henneh and Amu (82) indicated that creative arts such as music, dance, and movies play a significant role in the promotion of psychological well-being. In the case of internal migrants, the adoption of creative art like movies or comedy clips plays a crucial role in their lives in mitigating the various challenges they face as they are used as ways of replenishing the emotional and cognitive exhaustion, they experience (83) in job stress recovery through adaptive self-regulatory strategies (23). Seeking social support from others in sharing their lived experiences with their friends fosters social capital among fellow Kayayei that serves as an important social resource in times of difficulty (84).

Religion was found to play a major role as a coping strategy for most Kayayei in dealing with their daily working and living experiences and challenges. Their internalized religious beliefs and prayers help them to cope with stressful situations and bring hope and confidence that they will be able to overcome their difficulties. Religious coping strategies used among women were found to be protective against depressive symptoms (85). The study participants showed tolerance to suffering while their faith helped to make their challenges understandable and bearable (86). The finding of this study that most of the Kayayei adopted prayer as an essential coping strategy for their daily lived experiences was not surprising as religion plays a significant role in Ghanaian society (87).

In this study, the Kayayei demonstrated tremendous resilience in the face of the challenges they face. Their determination to continue trying to make a living for themselves and their families despite the daily adversity they experience and to remain hopeful for a better future seem to serve as inner resources that buffer their well-being (88). The Kayayei, view migration as essential in providing them with the financial and material gains that they lacked before migrating and are therefore determined to achieve this goal (89). The determination, motivation and goal-directedness displayed by the Kayayei in this study seemed to enable them to persevere and remain hopeful that their lives will soon change for the better despite the difficulties they experience. Positive emotions such as hope and optimism enhance resilience by expanding one’s range of thoughts, and actions for better coping and problem-solving as well as greater well-being as suggested by the broaden-and-build theory of positive emotions by Fredrickson (90).

However, maladaptive coping by the Kayayei in the form of self-medication for physical pain as outlined above and avoidance coping, e.g., drug use was also found. The physical and mental job demands of the Kayayei, just like other stressful jobs, predispose them to the abuse of over-the-counter drugs (91). Another predisposing reason is that they can hardly afford to fall sick or feel weak and miss out on a day’s work. The practice of self-medication often leads to drug dependence and abuse (92) The lack of health information and education on the consequences of self-medication among the Kayayei could also be a driving force of their self-medication. it is therefore important for health authorities to provide the Kayayei with relevant health promotion information to better understand the impact of their physical work on their mental and physical health and in particular ways to manage the build-up of lactic acid in the muscles to get relief instead of their reliance on self-medication (93).

The findings of the present study suggest the urgent need to strengthen the available personal resources of the Kayayei to buffer the job demands in their informal and unregulated work context. The multi-level JD-R model with a self-regulation perspective outlined by Bakker and de Vries (23) is useful to understand the interplay of the job demands and resources necessary to support the health and wellbeing of the Kayayei. The model specifically addresses personal resources and alludes to elements of psychological capital, i.e., self-efficacy, hope, optimism and resilience as developed by Lufthans et al. (94). These personal resources are viewed to be motivational and to help employees reach their goals (23) as also evident in this study. The findings highlight the current coping strategies employed by the Kayayei and call for the strengthening of their agency and inner resources, i.e., psychological capital as well as other resources, e.g., networking and social support to draw from in times of difficulties.

## Strengths and limitations of the study

As we used a smaller sample size and the non-probability sampling method per the tenets of qualitative studies, our findings cannot be generalized to all the Kayayei in Ghana. However, the detailed description of our methods ensures that the study findings are credible.

## Conclusion

This Study explored the Lived experiences, health-related challenges and the associated coping mechanism of internal migrants (Kayayei) in Ghana: a qualitative study There was evidence of physical health challenges, including accidents, pain, and fatigue. The majority of the participants also lack decent accommodation coupled with mistreatment by their patrons and society in general. There also seemed to be mental health challenges such as stress, anxiety, and depression. The results indicated that the prevalent coping strategies used by participants are self-regulated practices of self-care and recreational activities to de-stress as well as drawing from their inner resources namely internalized religious beliefs, positive emotions like hope, optimism, resilience and a future orientation despite their difficulties. The findings of this study are an indication of the important role of religion in mitigating the impact of challenges in Ghanaian society. It is worth noting that due to the physical nature of the Kayayei work, many of the participants seemed to employ maladaptive coping strategies such as self-medication to cope with the daily physical pain they go through. The use of the JD-R model was useful in understanding the role of job demands and resources in the health and well-being of the Kayayei. There is a need to strengthen the psychological capital and the agency of the Kayayei as well as social support to mitigate the high job demands in their informal work arrangements. It is however essential for the government of Ghana, in collaboration with various stakeholders, to develop policies for protecting the rights of informal workers especially women. We also recommend that the government of Ghana must work with stakeholders like social welfare and the Kayayei Association to raise awareness about women’s rights, build their skills to increase their employment opportunities, and enhance their safety, health, and overall well-being. It is also important to ensure the networking of relevant stakeholders to work with women in the informal sector to provide support when needed.

## Data availability statement

The raw data supporting the conclusions of this article will be made available by the authors, without undue reservation.

## Ethics statement

The studies involving humans were approved by College of Humanities, University of KwaZulu Natal Research Ethics Committee in South Africa Ref: HSS/0404/018D. The studies were conducted in accordance with the local legislation and institutional requirements. The participants provided their written informed consent to participate in this study. Written informed consent was obtained from the individual(s) for the publication of any potentially identifiable images or data included in this article.

## Author contributions

JK: Conceptualization, Formal analysis, Investigation, Methodology, Resources, Writing – original draft, Writing – review & editing. EM: Conceptualization, Validation, Visualization, Writing – original draft, Writing – review & editing. AM-W: Conceptualization, Methodology, Resources, Supervision, Writing – original draft, Writing – review & editing.
